# Potential Roles of the WNT Signaling Pathway in Amyotrophic Lateral Sclerosis

**DOI:** 10.3390/cells10040839

**Published:** 2021-04-08

**Authors:** Xin Jiang, Yingjun Guan, Zhenhan Zhao, Fandi Meng, Xuemei Wang, Xueshuai Gao, Jinmeng Liu, Yanchun Chen, Fenghua Zhou, Shuanhu Zhou, Xin Wang

**Affiliations:** 1 Department of Histology and Embryology, Neurologic Disorders and Regenerative Repair Laboratory, Weifang Medical University, Weifang 261053, China; 18363978239@163.com (X.J.); jinqiuhupan@163.com (Z.Z.); 18800460605@163.com (F.M.); 18863662168@163.com (X.W.); 17852060651@163.com (X.G.); 18353687185@163.com (J.L.); cyc7907@163.com (Y.C.); zhoufh@wfmc.edu.cn (F.Z.); 2 Department of Pathology, Weifang Medical University, Weifang 261053, China; 3 Department of Orthopedic Surgery, Brigham and Women’s Hospital, Harvard Medical School, Boston, MA 02115, USA; szhou@bwh.harvard.edu; 4 Department of Neurosurgery, Brigham and Women’s Hospital, Harvard Medical School, Boston, MA 02115, USA

**Keywords:** amyotrophic lateral sclerosis, WNT signaling, motor neurons, glial cells, neuromuscular junctions

## Abstract

The WNT signaling pathway plays an important role in the physiological and pathophysiological processes of the central nervous system and the neurodegenerative disease amyotrophic lateral sclerosis (ALS). We reviewed the literature pertinent to WNT/β–catenin signaling in ALS from cellular studies, animal models, and human clinical trials. WNT, WNT receptors, and other components of the WNT signaling pathway are expressed in both ALS patients and transgenic mice, and are involved in the pathogenesis of ALS. Studies have shown that abnormal activation of the WNT/β–catenin signaling pathway is related to neuronal degeneration and glial cell proliferation. WNT/Ca^2+^ signaling is associated with the pro–inflammatory phenotype of microglia; data on the muscle skeletal receptor Tyr kinase receptor in superoxide dismutase–1–G93A mice indicate that gene therapy is necessary for successful treatment of ALS. The varying profiles of lipoprotein receptor–related protein 4 antibodies in different ethnic groups suggest that individual treatment and multifactorial personalized approaches may be necessary for effective ALS therapy. In conclusion, the WNT signaling pathway is important to the ALS disease process, making it a likely therapeutic target.

## 1. Introduction

Amyotrophic lateral sclerosis (ALS) is a neurodegenerative disorder of unknown etiology. According to the inherited gene, ALS is designated either familial or sporadic. Approximately 5–10% of cases of ALS are familial cases and 90–95% of ALS cases are sporadic, occurring randomly without a family history. Since 1990, at least 25 mutated genes have been identified in familial and sporadic ALS. They involve varied mechanisms, including protein homeostasis, altered RNA–binding proteins, and cytoskeletal proteins. They have included, for example, mutations in superoxide dismutase–1 (*SOD1*), forced expression of high levels of normal *TDP–43* (encodes for TAR DNA binding protein), and repeat expansions on chromosome 9 open reading frame 72 gene (*C9ORF72*) [1,2,3,4]. The pathophysiology of ALS is characterized by degeneration of the upper and lower motor neurons in the motor cortex, brain stem, and spinal cord. Though the neurons that innervate the eye and sphincter muscles are spared, degeneration of other neurons causes muscles to go out of control, and death occurs when respiratory muscles are involved [1]. In addition to degenerated motor neurons, glial cells also undergo pathological changes, and their dysfunction produces a toxic environment in the central nervous system (CNS). Alteration of glial cells led to a key hypothesis in the pathogenesis of ALS, a “dying–back” mechanism. Specifically, that the disease originates in the peripheral tissues, including neuromuscular junctions (NMJs) and axonal degeneration, and then leads to motor neuron death in a retrograde signaling cascade [5,6,7]. The “dying–back” theory is based on literature showing denervation at the NMJ early in the life of ALS transgenic mice. After the onset of distal axon degeneration, microglial and astrocytic activation are identified around motor neurons [7,8]. Those findings indicate that the degenerative changes of ALS involve the entire motor unit. In conclusion, the pathogenic mechanisms of ALS include loss of motor neurons, abnormal astrocytes, dysregulation of microglial cells and oligodendrocytes, and degenerative changes in axons and NMJs. We found that the WNT signaling pathway is involved in these processes and plays a key role in the pathogenesis of ALS, both in the peripheral nervous system and in the CNS.

## 2. An Overview of the WNT Signaling Pathway

WNT signaling is a conserved pathway in animal development. In 1982, Nusse and Varmus [9] first discovered the mouse *Wnt1* gene, originally named *Int1*, a proto–oncogene cloned from mouse breast cancer. To date, at least 19 WNT members (*WNT1*, *WNT2*, *WNT3A*, *WNT4*, *WNT5A*, *WNT8*, *WNT11*, etc.) have been found in humans and mice [10]. The *WNT* genes encode WNT proteins to activate the WNT signaling pathway, which comprises the β–catenin–dependent pathway (WNT/β–catenin pathway) and the β–catenin–independent pathway, previously known as the canonical and non–canonical WNT signaling pathways, respectively. The β–catenin–independent pathway includes both the WNT/Planar Cell Polarity (WNT/PCP) and the Wnt/Ca^2+^ pathways [10,11]. A combination of ligands and receptors mediates signaling pathways, but the specific combinatorial code remains unsolved. First, there are more than 19 different WNT ligands and 15 different receptors and co–receptors. Second, the three pathways are not independent, but instead form a complex signal network. The receptors of Wnt signaling include the seven–pass transmembrane receptors Frizzled1–10 (FZD1–10), the single–pass transmembrane co–receptors low–density lipoprotein receptor–related protein 5/6 (LRP5/6), low–density lipoprotein receptor–related protein 4 (LRP4) [12], receptor Tyr kinase–like orphan receptor1/2 (ROR1/2), muscle skeletal receptor Tyr kinase (MUSK), receptor Tyr kinase (RYK), protein Tyr kinase 7 (PTK7), and the proteoglycan family [10]. Although the specific combinatorial code remains unresolved, it is well known that WNT1, WNT3A, and WNT8 are predominantly involved in the WNT/β–catenin pathway, and WNT5A and WNT11 are more commonly encountered in the β–catenin–independent pathway [10,13].

### 2.1. WNT/β–Catenin Signaling

**Figure 1:**
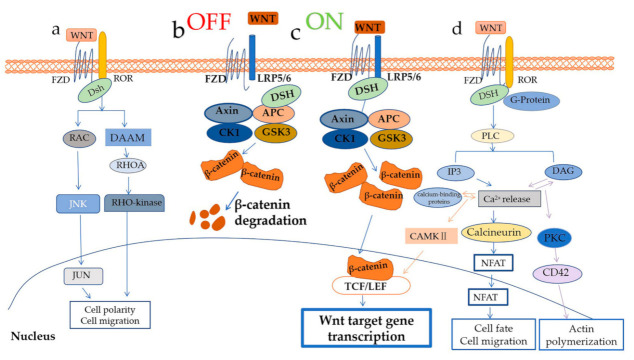
WNT signaling pathways. WNT regulates at least three distinct intracellular signaling pathways: (**a**) The WNT/Planar Cell Polarity (PCP) pathway. (**b**,**c**) the WNT/β–catenin pathway: (**b**) the OFF state of the WNT/β–catenin pathway, absent WNTs stimulation; (**c**) the ON state of WNT/β–catenin pathway, when the WNT ligands couple to receptors. (**d**) the WNT/Ca^2+^ pathway. Please see text for other details.

The crucial mediator of the WNT/β–catenin pathway (Figure 1b,c) is β–catenin protein [13], which is also a central mediator of cell-cell interaction. In the latter role, β–catenin protein protects the cadherin cytoplasmic domain from proteolysis and maintains adherent junctions between cells, preventing the cadherin cytoplasmic domain from undergoing proteolysis [14]. In the WNT/β–catenin pathway, β–catenin protein is protected by an association of WNT ligands and receptors, and the destroyer of β–catenin is a destruction complex consisting of adenomatous polyposis coli gene product (APC), scaffolding protein AXIN, casein kinase 1 (CK1), and glycogen synthase kinase 3 (GSK3). GSK3 includes both GSK3α and GSK3β subtypes [10]. Binding of WNT ligands and FZD receptors and co–receptors LRP5 and LRP6 can active WNT/β–catenin signaling (WNT–on state Figure 1c).

The key event after binding of the WNT ligand to its receptor is GSK3β– and CK1γ–mediated phosphorylation of LRP6, which allows LRP6 to recruit AXIN [15]. Phosphorylation of LRP6 also requires the binding of Dishevelled (DSH) to FZD, which enables interaction of LRP6 and AXIN, in turn leading to the degradation of the destruction complex [16]. The degraded destruction complex prevents β–catenin phosphorylation by GSK–3β and subsequent degradation [17]. As a result, β–catenin proteins are increased in the cytoplasm, and accumulated β–catenin proteins translocate into the nuclei, where they regulate target gene expression. The expression of target genes depends on the cooperation between β–catenin and transcription factors. The major transcription factors of WNT/β–catenin signaling are T–cell factor and lymphoid enhancer factor (TCF/LEF), which are multifunctional proteins that specify gene expression with sequence–specific DNA–binding and context–dependent interactions [18]. Target genes are involved in proliferation, apoptosis, cell cycle, differentiation, metabolism, inflammation, immune response, and cell adhesion [19].

In the absence of WNTs (WNT–off state Figure 1b), AXIN serves as a scaffold for GSK3, CK1α, and β–catenin. β–catenin is then phosphorylated in the N–terminal domain, with CK1α phosphorylated at serine 45, followed by GSK3 phosphorylated at serine 41, serine 37, and serine 33. The E3 ubiquitin ligase β–TrCP causes ubiquitination and degradation of β–catenin by recognizing binding sites formed by phosphorylation of serine 33 and 37 [20].

### 2.2. WNT/PCP Pathway and WNT/Ca^2+^ Pathway

The WNT/PCP pathway (Figure 1c) regulates tissue patterning and morphogenesis, for example, cell movement during vertebrate gastrulation. In the WNT/PCP pathway, the binding of WNT ligands, FZD receptors, and ROR co–receptors activate GTPases RAC and RHOA via DSH–associated activator of morphogenesis (DAAM) proteins. They activate RHO kinase (ROCK) and JUN N terminal kinase (JNK), respectively, leading to gene expression [10,13,21].

The WNT/Ca^2+^ (Figure 1d) pathway is another β–catenin–independent signaling pathway; it controls intracellular calcium levels through regulating calcium release from the endoplasmic reticulum, in addition to activating protein kinase C (PKC) and calcineurin [22,23]. In this case, WNTs associated with FZD receptors activate DSH via G–proteins, which in turn activate phospholipase C (PLC). Consequently, diacylglycerol (DAG) and inositol–1,4,5–trisphosphate (Ins (1,4,5) P3) production is stimulated, leading to Ca^2+^ release from the endoplasmic reticulum [10]. The increased Ca^2+^ interacts with calcium–binding proteins to activate calcium calmodulin–mediated kinase II (CAMKII), which along with DAG activates PKC [22,24]. CaMKII and PKC activate various transcription factors to regulate target gene expression. Calcineurin activated by the released Ca^2+^ activates the nuclear factor of activated T cells (NFAT), which in turn regulates the transcription of genes that control cell fate and cell migration [10].

## 3. Alteration of WNT Signaling in ALS

Under physiological conditions, WNT/β–catenin signaling drives the differentiation of neural precursor cells into neurons. Blockade of signaling inhibits neuronal differentiation of neural precursor cells in in vitro and in vivo models [25]. WNT signaling also modulates hippocampal neurogenesis and enhances the proliferation of neural stem cells as well as synaptic stability and plasticity [26]. These novel findings indicate that focusing on WNT signaling may yield some improvement in ALS treatment. Indeed, the mRNA or protein expression of WNTs, receptors, co–receptors, and modulators are altered in ALS post–mortem spinal cord tissue and ALS transgenic mice. Our group focuses on the study of WNT signaling in the pathology of ALS in the SOD1–G93A mouse model [27,28,29,30,31], the most frequently used mouse line in the study of ALS. Animals in this line overexpress the mutant SOD and develop clinical and neuropathological features resembling human ALS [4,32,33]. We found that WNT/β–catenin signaling is activated in SOD1–G93A transgenic mice.

### 3.1. Activated WNT/β–Catenin Signaling in ALS

Upregulation of the components of WNT/β–catenin signaling has been detected in ALS (Table 1). In the spinal cords of SOD1–G93A mice, we found elevated expression of WNT1, WNT2, WNT3A, and WNT7A (Table 1), which are the upstream effectors of WNT/β–catenin signaling and activate β–catenin protein [29,30,31]. GSK–3β is one component of the destruction complex (seen in Figure 1b,c), which reduces the stability of β–catenin in the cytoplasm. Its activity can be inhibited by phosphorylation of GSK–3β Ser 9. Our study found that the expression of GSK–3β is unchanged, but the expression of phospho–GSK–3β (Ser 9) is increased (Table 1) in SOD1–G93A mice [30]. The changes support the stability of β–catenin in the cytoplasm. The β–catenin protein, a key protein in WNT/β–catenin signaling, translocates from the cytoplasm to the nucleus in the later stages of SOD1–G93A mice. Western blot analysis also confirmed an increase in nuclear β–catenin protein (Table 1) [29]. The nuclear translocation of β–catenin is an important sign of activation of the WNT/β–catenin signaling pathway. Pinto et al. [34] also found markedly increased β–catenin accumulation in ALS motor neurons, including increased supramolecular structures in SOD1–G93A mice and the NSC34 motor neuron–like cell line. Thus, based on the evidence outlined above, WNT/β–catenin signaling is hyperactivated in ALS transgenic mice, but it is unclear whether this alteration is beneficial or harmful.

### 3.2. Alteration of the WNT/PCP and WNT/Ca^2+^ Pathways in ALS

WNT5A, FZD2, and FZD5 have proven important molecules in the WNT/Ca^2+^ pathway. The association of WNT5A and FZD2 increases intracellular Ca^2+^ accumulation and activated CAMKII [35]. The interaction of FZD5 and WNT5A regulates the inflammatory response in macrophages through the WNT/Ca^2+^ pathway [36]. The expression of these molecules is altered in both ALS patients and transgenic mice (Table 1). For example, the number of FZD2–positive astrocytes are significantly elevated in ALS transgenic mice and ALS patients [28,37]. Primary cultivation of astrocytes from ALS transgenic mice also reveals upregulated FZD2 expression [28]. Moreover, FZD2 positive astrocytes are distributed in regions where neurons are lost [37]. WNT5A acts as the ligand of FZD2, whose expression is parallel with that of FZD2 and located in the same affected areas [28,37]. The immunoreactivity of WNT5A is increased in reactive FZD2–positive astrocytes [37], and in pEGFP–G93A–SOD1 plasmid transfected NSC34 cells the expression of WNT5A is downregulated [28]. Expression of the FZD5 receptor protein is clearly upregulated throughout the progression of symptoms and co–localized with neurons, but not astrocytes, in the spinal cords of SOD1–G93A mice. Under physiological conditions, the FZD5 receptor is necessary for neuronal survival in the nucleus of the thalamus [38] and synaptic physiology, being localized at both pre– and post–synaptic sites [39]. The cellular distribution of FZD5 indicates its relationship with neuron survival and function [40].

WNT4 regulates many biological processes through the WNT/PCP pathway, including NMJ formation and murine hematopoietic progenitor cell expansion [41,42]. WNT4 protein is upregulated in the pathogenesis of ALS (Table 1). The expression of WNT4 is increased in mature astrocytes but decreased in neurons, especially in the final stages of ALS [27].

According to the above description, the β–catenin–independent pathway is involved in the neurodegeneration of ALS. However, the altered expression of WNT ligands and receptors does not adequately represent the changes in non–canonical WNT signaling, and it is necessary to further confirm how those altered components affect the death of neurons and the proliferation of glial cells.

## 4. Dysregulated WNT/β–Catenin Signaling in Neurons and Glial Cells

### 4.1. Extensive β–Catenin Accumulation in Motor Neurons

**Figure 2:**
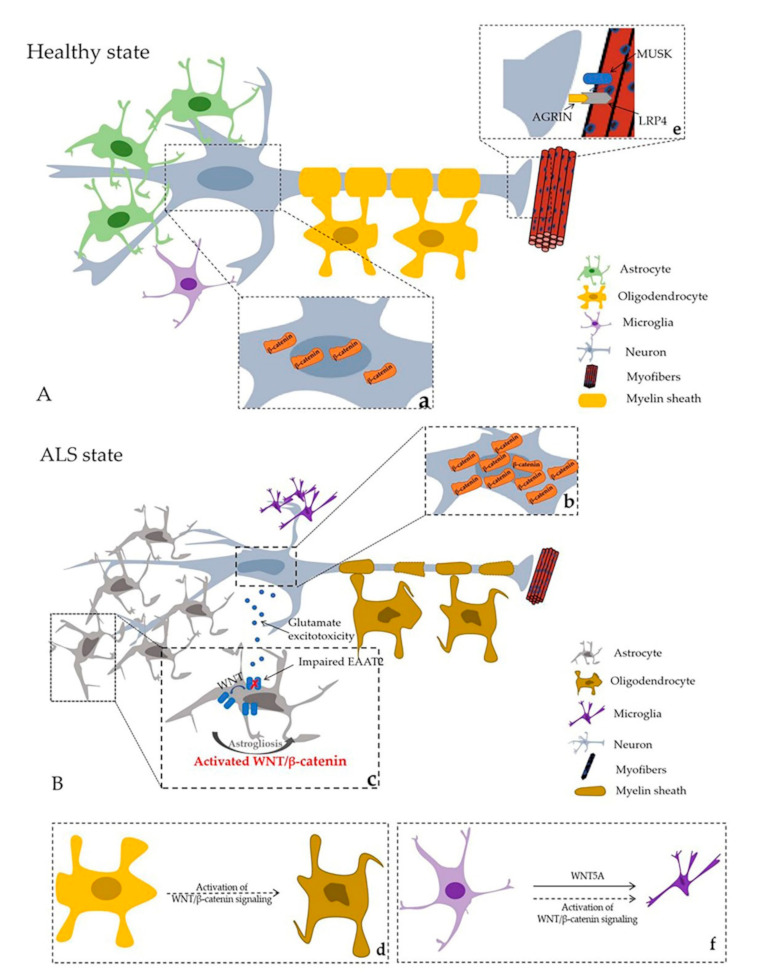
Healthy state and ALS state in central nervous system (CNS) and neuromuscular junction (NMJ). (**A**) Healthy state; (**B**) ALS state; a. and b represent β–catenin distribution in healthy and ALS states, respectively; **a**. Normal β–catenin distribution in motor neurons. **b**. Extensive β–catenin accumulates in the cytoplasm in the motor neuron cell model in vitro. β–catenin protein nucleus translocation can be observed in the later stage of ALS in SOD1–G93A mice. **c**. Astrogliosis and molecular and functional abnormalities of astrocytes in ALS. **d**. Axon damage and oligodendrocyte degeneration. **e**. AGRIN–LRP4–MUSK complex promote stability of postsynaptic differentiation. **f**. WNT signaling and proinflammatory of microglia (black arrows represent phenomena in ALS; dashed arrows illustrate hypothetical modes in ALS).

**Figure 3:**
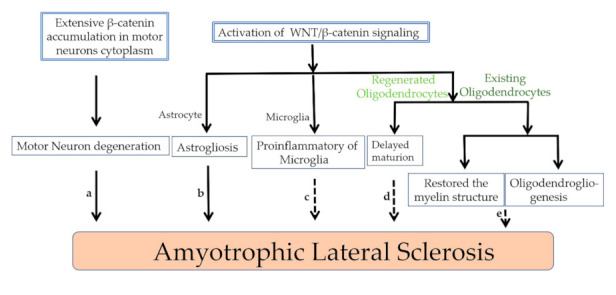
WNT/β–catenin signaling and β–catenin protein contribute to the onset of ALS. **a**. The extensive accumulation of β-catenin in motor neurons results in the degeneration of motor neurons in ALS. **b**. Activation of WNT/β–catenin signaling affects astrogliosis in ALS. **c**. Activation of WNT/β–catenin signaling promotes the transformation of microglia to proinflammatory phenotype. **d**. Activation of WNT/β–catenin signaling delays the maturation of regenerated oligodendrocytes. **e**. For the existing oligodendrocytes, activation of WNT/β–catenin signaling promotes the restored of degenerative myelin structure and oligodendrogliogenesis. Black arrows represent confirmed phenomena in ALS; dashed arrows illustrate hypothetical models in ALS.

Cytosolic β–catenin proteins aggregate abnormally in the ALS in vitro model, which is constructed using motor neuron–like NSC34 cells that stably express G93A mutant SOD1 [34,50] (Figure 2Aa,Bb and Figure 3a). Abnormal β–catenin inmutated hSOD1 NSC34 cells does not co–localize with common protein aggregates cleared by degradative pathways [34], because the abnormal β–catenin does not form insoluble protein aggregates and may assemble as a three–dimensional structure of amorphous aggregates, in which monomers are assembled randomly. Moreover, the abnormal presence of peripheral β–catenin structures is associated with impaired cell–cell interaction and decreased neuronal differentiation [34].

It is surprising that disassembly of accumulated β–catenin structures can rescue the morphological differentiation of ALS–like motor neurons [34]. In the neuronal model of Huntington’s disease (HD) in drosophila, reduced β–catenin levels have a neuroprotective effect and extend the lifespan of HD flies [51]. On the other hand, loss of WNT/β–catenin signaling makes neurons more sensitive to Aβ–induced apoptosis and improves disorders of memory and spatial learning [52]. The above description suggests the presence of various abnormal β–catenin in different diseases.

### 4.2. Activation of WNT/β–Catenin Signaling and Astrogliosis

Astrocytes are the most abundant cells in the CNS. They maintain ion homeostasis, regulate neurotransmitter circulation, and provide metabolic support for surrounding neurons to protect them from oxidative damage and support their survival [53,54]. Thus, dysregulation of astrocytes produces an environment toxic to neurons. When the CNS suffers insults, such as neurodegeneration, astrocytes show astrogliosis (Figure 2A,Bb and Figure 3d), characterized by increased glial fibrillary acidic protein (GFAP) [55,56]. Astrogliosis sometimes yields a net benefit, but under certain circumstances can result in damage [57]. In both animal models and patients, astrogliosis is a morphological feature in various areas of the brain during ALS pathogenesis [58,59,60]. Degeneration of upper and lower motor neurons is accompanied by astrogliosis [61]. We found several components of WNT signaling, for example, WNT1, WNT2, WNT3A, WNT4, and WNT7A, the core component of β–catenin, and receptors FZD1 located in the increased astrocytes [27,28,29,30,31]. In the developing spinal cord, WNT/β–catenin signaling is related to the generation of astrocytes. Activation of WNT/β–catenin signaling inhibits the differentiation of astrocytes from neural progenitor cells (NPCs), but inhibition of WNT/β–catenin signaling activates the differentiation of astrocytes from NPCs [62]. In addition, transcriptomic changes are found in the cortical astroglia of an ALS transgenic mice model; over 1000 genes are dysregulated. Several of the most highly upregulated genes are related to the WNT pathway, especially the WNT/β–catenin pathway. Coiled–coil domain containing 85B was the gene most strongly upregulated; it is a nuclear transcriptional repressor and inhibits the function of β–catenin in a p53–dependent manner [63].

In addition to abnormal morphological changes, astrocytes also show molecular and functional abnormalities in ALS, characterized by reduced and impaired excitatory amino acid transporter EAAT2 (glutamate transporter 1, GLT–1 in rodents). This leads to increased levels of the excitatory amino acids glutamate and aspartate and their metabolites in cerebrospinal fluid (Figure 2Bc) [64,65], which can cause prolonged exposure of glutamate receptors to a high concentration of glutamate, leading to their dysregulated activation (a.k.a. glutamate excitotoxicity) [58]. In ALS, motor neurons are susceptible to glutamate excitotoxicity. Under physiologic conditions, extracellular glutamate is maintained in a low concentration range, which is inadequate to activate glutamate receptors. Maintenance of the normal glutamate concentration range depends on the clearance of glutamate transporters. In humans, there are five Na+–dependent glutamate transporters, referred to as excitatory amino acid transporters 1–5 (EAAT 1–5). The glial glutamate transporter EAAT2 is located mainly on astrocytes and accounts for 1% of total brain proteins, suggesting that EAAT2 is responsible for the largest glutamate transport in the CNS [66]. In glioma cells, WNT signaling has been demonstrated to induce EAAT2 expression [66]. In Parkinson’s disease, the neuroprotective effect of astrocytes on dopaminergic neurons is mediated by WNT1 upregulation of EAAT2 expression [67].

Astrocytes overexpressing WNT1 can promote and protect EAAT2 expression, and glutamate concentration in culture medium decreases after co–culture with neurons (Figure 2Bc) [67]. The expression of EAAT2 is increased in ALS astrocytes. SOD1–G93A mice treated with ceftriaxone, a drug that increases EAAT2 protein expression before ALS onset, produced decreased motor neuron loss after two weeks’ treatment, but the effects disappeared in a phase 3 clinical trial [68,69]. In addition to EAAT2 regulating glutamate levels, astrocytic LRP4 also modulates glutamate release [70]. Ablation of LRP4 specifically in astrocytes reduced the frequency of miniature excitatory postsynaptic currents (mEPSCs) and the probability of glutamate release [70]. LRP4 mutant astrocytes impair glutamatergic transmission. Glutamatergic transmission in LRP4 mutant mice is likely suppressed by A1 receptor activation by adenosine, a product of ATP hydrolysis, offering insight into the interaction between neurons and astrocytes for synaptic homeostasis and/or plasticity [70].

### 4.3. Activation of WNT/β–Catenin Degenerated Oligodendrocytes

Oligodendrocytes are affected by ALS. In SOD1–G93A mice, oligodendrocytes have degenerated extensively long before motor neurons begin to degenerate [71,72]. Oligodendrocytes generate and maintain the myelin sheath insulating the axon [73] as well as providing neurons with metabolic support [74]. Abnormalities in either of these two functions are important causes of several neurodegenerative diseases [73,74].

Oligodendrocytes are the progeny of oligodendrocyte progenitor cells. During embryonic development of the CNS, neural stem cells (NSCs) generate oligodendrocyte progenitor cells (NG^2+^ glial cells), which first differentiate into immature oligodendrocytes and then mature into functional myelinating cells. Oligodendrocytes remain in a stable myelinating state during adult life. When they encounter CNS injury, NG^2+^ oligodendrocyte precursor cells increase proliferation and differentiate again into oligodendrocytes. However, these newly formed oligodendrocytes show abnormal functions, for example, impaired myelin repair efficiency [75], resulting in immature myelin sheaths, swollen electron–dense myelin sheaths, and non–myelinating oligodendrocytes [72]. This indicates that the dysmaturity of oligodendrocytes may be affected by some unknown factors.

Increasing evidence shows that WNT/β–catenin signaling plays a vital role in oligodendrocyte development under physiological or pathological conditions (Figure 2Bf). (i) In the dorsal subependymal zone, in vivo activation of WNT/β–catenin signaling by WNT3A treatment leads to an increase in oligodendrocyte proliferation [76]. (ii) In vivo inhibition of GSK3β stimulates oligodendrocyte progenitor cell proliferation by stimulating nuclear translocation of β–catenin to the lateral ventricle of postnatal mice [77]. (iii) The inhibition of GSK3β also restored the myelin structure after chemically induced demyelination following nerve crush injury [77,78], but WNT/β–catenin signaling prevented further differentiation of oligodendrocytes from progenitors to immature oligodendrocytes during spinal cord development [79]. Finally, the activation of WNT/β–catenin signaling inhibits oligodendrocyte maturation [80,81], but it does not affect oligodendrocyte precursor cells, resulting only in delayed mature oligodendrocytes, myelin protein, and myelinated axons during development [80].

We conclude that the effects of WNT/β–catenin signaling on oligodendrocytes in injured CNS can be divided into two phases. First, the activation of WNT/β–catenin signaling through inhibition of GSK3β has a positive effect on existing oligodendrocytes (Figure 3e). Second, WNT/β–catenin signaling does harm to mature oligodendrocytes during the regeneration process under pathological conditions (Figure 3d).

### 4.4. WNT5A and Activation of WNT/β–Catenin Signaling Induced Proinflammatory Microglia

One of the hallmarks of ALS is neuroinflammation, which is characterized by microgliosis. Analysis of postmortem ALS patient CNS tissue detected extensively increased microgliosis [82,83]. Activated microglia release many inflammatory cytokines and chemokines [84,85]. Studies in the mSOD1 mice model revealed that eliminating microglial activation delays the progress of disease [86]. Activation of proinflammatory microglia occurs in the spinal cord prior to the onset of clinical disease, increases with disease progression, and persists until death of SOD1–G93A mice [87]. However, the molecular cascade of proinflammatory of microglia remains unsolved.

NF–κB is a transcription factor mainly regulating immune responses and inflammation. Astrocytic NF–κB is a master regulator of microglia expansion, and WNT5A is an important mediator in this process. WNT5A is secreted by astrocytes in SOD1–G93A mice, induces microglial proliferation, and is the effector of NF–κB [88]. WNT5A induces pro–inflammatory transformation of primary microglia in mice through increased microglial proliferation [89]. Inhibition of WNT5A secretion reduces microglial proliferation and cell density [88]. This line of evidence demonstrates that astrocytic WNT5A is an important regulator of microglial expansion and immune response in ALS.

Research in patients and a transgenic animal model of Alzheimer’s disease revealed that β–catenin levels are elevated in active microglia and that WNT3A stimulation increases the release of proinflammatory cytokines in microglia [90]. TWS119, an activator of the WNT/β–catenin pathway, facilitates neurological recovery after chronic cerebral ischemia via modulating microglia towards an anti–inflammatory phenotype [91] (Figure 3c).

## 5. Receptor RYK and Axon Dysregulation

Axonal injury tends to occur in the early stages of ALS (Figure 2Bd) [92]. WNTs act as cues for axon growth in the CNS. Several WNT ligands (WNT7A, WNT7B, WNT4, and WNT5A) control the appropriate turning of anterior–to–posterior axons through FZD3. Receptor RYK promotes the growth of descending corticospinal tract axons along the anterior–posterior axis of the spinal cord, and then WNT5A guides the growth of corticospinal tract axons along the spinal cord [93].

WNTs that reappear after spinal cord injury lose these beneficial properties and instead restrict motor and sensory axon regeneration [93]. Expression of WNT5A is apparently upregulated in the spinal cord of ALS patients and mice [28,37]. RYK expression is increased in motor axons and motor neurons of ALS mice throughout the progression of the disease [94]. Inhibition of WNT–RYK signaling promotes sprouting of collaterals from the spared spinal cord [95]. Those changes may ameliorate axonal degeneration in the progress of ALS.

## 6. WNT Proteins and NMJs in the Progress of ALS

### 6.1. NMJs in ALS

The NMJ is the synapse between the skeletal muscle and the spinal motor neuron, critical for communication between nerves and muscle. Loss of an effective connection in the progress of ALS results in complete muscle paralysis; disruptions of the neuromuscular junctions are an early pathologic sign of ALS [7]. Therapeutic interventions that preserve neuromuscular synapses may slow the loss of motor function and improve the quality of life for patients. If denervation of the NMJ results in the loss of motor neurons, this therapeutic intervention may be worthwhile to delay the death of motor neurons and extend survival.

### 6.2. WNT Proteins and Receptors Contribute to Neuromuscular Junction Formation

The essential medium of neuromuscular transmission is acetylcholine (ACh), released from presynaptic terminals [96]. Dense acetylcholine receptor (AChR) clusters ensure efficient neuromuscular signal transmission [97]. A hallmark of the inductive events of NMJ formation is the restrictive spatial patterning of AChRs in synaptic sites [98]. Agrin is a large glycoprotein released by motor nerve terminals that combines with LRP4 and activates MUSK, forming the Agrin–LRP4–MUSK complex to stabilize postsynaptic differentiation (Figure 2Ae) [99].

WNT ligands play important roles in the disease process of ALS. Current research indicates that among the 19 WNTs in mammals, WNT2, WNT3A, WNT4, WNT6, WNT7B, WNT9A, and WNT11 directly interact with MUSK and WNT4, WNT9A, and WNT11 to regulate AChR clustering in muscle cells [100]. Studies in mice models and cell culture demonstrate that WNT4 and WNT11 enhance clustering of AChR and motor axon outgrowth. Both WNT4 and WNT11 not only activate the WNT/β–catenin pathway but also increase AChR mRNA levels and AChR clustering. By contrast, loss of WNT11 or inactivated WNT/β–catenin signaling decreases clustered AChR and motor nerve terminal branching. Mammalian NMJ is formed through WNT/β–catenin and WNT/PCP signaling, mediated by WNT4 and WNT11 [100]. WNT3 independently enhances AChR clusters during the process of motoneuron–muscle innervation [101]. However, WNT3A inhibits AChR clusters in cultured muscle cells through downregulating the WNT/β–catenin pathway [102].

### 6.3. Frizzled Related Protein (FRZB) and Receptor MUSK in NMJs and Skeletal Muscle: Early ALS Diagnosis

NMJ and skeletal muscle are the earliest sites of the effects of ALS, and it is of prime importance to identify biomarkers for them in the diagnosis of ALS. FRZB, the WNT antagonist, is a molecular sign of muscle denervation in ALS and begins to increase in skeletal muscle in the early pre–symptomatic period. This discovery offers benefits for diagnosing and tracking disease progression [46].

The receptor MUSK is also the focus of NMJ exploration in ALS. In terms of the important role of MUSK, many studies have investigated whether increasing MUSK expression might benefit ALS patients. Increased expression of MUSK in SOD1–G93A mice (3–fold higher than in control mice) is sufficient to sustain neuromuscular synapses and improve muscle function for over 30 days [103]. Treatment with MUSK agonist antibodies preserved innervation of the neuromuscular junction longer than in control–treated mice, but offered no substantial functional benefit in SOD1–G93A mice [104,105]. Drug therapy alone does not bring clinical benefits, further suggesting that gene therapy is essential to the treatment of ALS.

### 6.4. LRP4 Autoantibody Detection in ALS Cases

Sera from Greek and Italian patients with sporadic ALS contain more LRP4 autoantibodies (23.4%) than sera from patients with other neurological diseases (3.6%) or from healthy controls [48]. Positive LRP4 autoantibodies also exist in cerebrospinal fluid samples from six of seven tested LRP4 antibody–seropositive patients. The autoantibody titer roughly corresponds with the clinical condition (stable condition correlates with decreased antibody titer, worsening condition correlates with increased antibody titer) [48]. In a study of Chinese ALS patients, 5.4% (3 of 56) patients were positive for LRP4 autoantibodies, which is lower Greek and Italian patients. The anti–LRP4 antibody was found to last 16 months in Chinese ALS patients, but 36 months in Greek and Italian patients, it is possible that anti–LRP4 antibodies may be related to ethnicity [49]. However, the positive LRP4 autoantibodies appears relatively late and is not an ideal indicator for early diagnosis. In conclusion, the worse the condition, the higher the titer of anti–LRP4 antibodies in ALS patients.

## 7. Special Case in ALS–Spared Extraocular Muscle and WNT Ligands

Until late in the disease, neurons that innervate the eyes are spared in ALS, including extraocular muscles and their motor neurons [1,47]. This contrasts sharply with the extreme atrophy of muscles innervated by other cranial nerves and limb muscles. One study exploring the differential effects of ALS in extraocular muscles (EOM) and limb muscles found different expression of WNTs and β–catenin, with the strongest difference in the NMJ [47]. The expression of WNT1, WNT3A, and WNT7A is decreased in axons of the human limb muscles. WNT1 and WNT3A expression in myofibers is also decreased, but WNT7A is elevated. WNT5A expression in all the axons within the EOM and limb muscles is not significantly changed. In ALS transgenic mice at the terminal stage, WNT1, WNT3A, WNT5A, and WNT7A are decreased in the neuromuscular junctions; but these changes in WNT ligands were not significantly associated with β–catenin–positive myofibers [47].

## 8. Conclusions and Remarks

As we have discussed in this review, evidence indicates that WNTs, WNT receptors, and other core components of the WNT pathways participate in the dysfunction of the entire motor unit, from axon terminal denervation to the death of motor neurons. The abnormal expression of WNT ligands [47] at the EOM and limb in ALS further illustrates the special role of WNTs. First, abnormal activation of WNT/β–catenin signaling leads to the degeneration of neurons and the proliferation of glial cells. The WNT/PCP pathways (WNT/Ca^2+,^ and WNT/β–catenin signaling pathways) do not exist independently, increasing the complexity of this signal network. Because many molecules involved in WNT signaling are altered in ALS, it is worth exploring the mode of action of WNT signaling in the process of ALS. Second, data accumulated regarding the MUSK receptor in SOD1–G93A mice indicates that gene therapy is essential to treatment of ALS. Third, the varying profiles of LRP4 antibodies in different ethnic groups suggest significant individual differences in the disease process of ALS. This, individual treatment and multifactorial personalized approaches are necessary. Full characterization of the relationship between ALS and WNT signaling will greatly improve our understanding of the mechanisms of ALS and better inform our exploration of novel therapies.

## Figures and Tables

**Table 1:** Changes in levels of WNT signaling components in human ALS- and SOD1–G93A transgenic mouse and motor neuron.

Methods	Research Subject		Inhibitor and Antagonist	WNT Ligands	Receptors	Downstream Molecules
Gene Expression Profiling PCR Array	SOD1–G93A transgenic mouse spinal cords [27]	95 d		↑ *Wnt1, Wnt3a, Wnt7b, Wnt8a*	↑ *Fzd3*	↑ *Fosl1, Frat1* ↓ *Myc, T, Tcf7*
		108 d	↑ *Wif1* and *sFrp4*	↑ *Wnt4* ↓ *Wnt16*, *Wnt8b*		↑ *Fgf4, Fosl1, Sfrp4*, ↓ *Csnk1a1, Frat1, FrzbLef1, Nkd1, Pitx2, Sfrp1, T, Tle2*
		122 d		↑ *Wnt10a*, *Wnt11, Wnt16*, *Wnt2*, *Wnt3*, *Wnt4, Wnt5a, Wnt5b, Wnt7a, Wnt7b, Wnt9a*	↑ *Fzd1, Fzd2, Fzd3*, *Fzd4, Fzd5, Fzd6, Fzd7, Fzd8, Lrp5*	↑ *Btrc, Ccnd1/2/3, Csnk1a–1, Ctnnb1, Dvl1, Ep300, Fbxw4/11, Fosl1, Frzb, Jun*, *Myc, Nlk, Pitx2, Ppp2r5d, Rhou, Senp2, Sfrp4*, *Slc9a3r1, Sox17, T, Tcf3*, *Wif1* ↓ *Tcf7*
Gene Expression Profiling PCR Array	ALS human spinal cords [37]		↑ *sFRP3*	↑ *WNT3, WNT4, WNT2B, WNT5A*	↑ *FZD2*, and *FZD8, FZD3, LRP5*,	
Gene Expression Profiling PCR Array	SOD1–G93A transgenic mouse Skeletal Muscle [43]	40 d				↑ *Prkx, Dner*
		80 d			↓ *Fzd2*	↓ *Cd44*
RNA sequencing	SOD1 mutant motor neurons(iPSC) [44]				↑ *Fzd2*	↑ *Lef, Tcf7l2* *β–catenin*
RNA sequencing	hSOD1–G93A mutant motor neurons (NSC34 cell) [45]					↑ *Cltc, Plcb3, Plec, Psmd3, Ruvbl1*
Immunofluorescence, Western blot, RT–PCR	astrocytes in SOD1–G93A transgenic mouse spinal cords			↑ WNT2A, WNT7A [30] WNT3A [29] WNT5A [28]	↑ FZD2 [28]	↑ nuclear β–catenin [29]
Immunofluorescence	astrocytes in ALS human spinal cords [37]			↑ WNT5A	↑ FZD2	
Immunofluorescence, Western blot, RT–PCR	neurons in SOD1–G93A transgenic mouse spinal cords			↓ WNT3A [29] WNT5A [28]	↑ FZD5 [40]	↑ nuclear β–catenin, Phospho–GSK–3β (Ser 9) [29]
Immunofluorescence	hSOD1–G93A mutant motor neurons (NSC34 cell) [34]					↑ cytoplasm β–catenin
RNA sequencing, Western blot	Skeletal muscle and the neuromuscular junction in ALS human and SOD1–G93A mouse [46]		↑ FRZB			↑ β–catenin
Immunofluorescence [47]	limb muscles in ALS human			↓ WNT1, WNT3A, WNT7A		↑ β–catenin
	myofiber in ALS human			↑ WNT7A ↓ WNT1, WNT3A,		
	neuromuscular junctions in SOD1–G93A mouse			↓ WNT1, WNT3A, WNT5A WNT7A		
	extraocular muscles in ALS human and SOD1–G93A mouse					↑ β–catenin
cell–based assay and radio– immunoassay	sera from sporadic ALS patients [48,49]				↑ LRP4	

↑: increase, ↓: decrease; Btrc, Beta–transducin repeat containing protein; Ccnd1/2/3, Cyclin D1/2/3; snk1a1, Casein kinase 1, alpha 1; Ctnnb1, Catenin (cadherin associated protein), beta 1; Dner, Delta Notch ligand/receptor; Dvl1, Dishevelled, dsh homolog 1 (Drosophila); Ep300,E1A binding protein p300; Fbxw4/11, F–box and WD–40 domain protein 11; Fgf4, Fibroblast growth factor 4; Fosl1, Fos–like antigen 1; Frat1, Frequently rearranged in advanced T–cell; Frzb, Frizzled–related protein; FRZB, Frizzled Related Protein; Jun, Jun oncogene; Lef1, Ly–phoid enhancer binding factor 1; Myc, Myelocytomatosis oncogene; Nkd1, Naked cuticle 1 homolog (Drosophila); Nlk, Nemo like kinase; Pitx2, Paired–like homeodomain transcription factor 2; Prkx, protein kinase X gene; Ppp2r5d, Protein phosphatase 2, regulatory subunit B (B56), delta isoform; iPSC, induced pluripotent stem cells; Rhou, Ras homolog gene family, member U; Senp2, SUMO/sentrin specific peptidase 2; sFRP13//4, Secreted frizzled–related protein 4; Slc9a3r1, Solute carrier family 9 (sodium/hydrogen exchanger), member 3 regulator 1; Sox17, SRY–box containing gene 17; RUVBL1, RuvB Like AAA ATPase 1; T, Brachyury; Tcf3/7, Transcription factor 7; Tle2, Transducin–like enhancer of split 2; Wif1, WNT inhibitory factor 1.

## Abbreviations

ACh	Acetylcholine
AChR	acetylcholine receptor
ALS	amyotrophic lateral sclerosis
APC	adenomatous polyposis coli
ATF2	activating transcription factor 2
CK1	casein kinase 1
CNS	central nervous system
C9orf72	chromosome 9 open reading frame 72 gene
CAMKII	calcium calmodulin mediated kinase II
Dsh	Dishevelled
DAG	Diacylglycerol
EOM	extraocular muscles
EAAT 1–5	excitatory amino acid transporter 1–5
FRZBFZD 1–10	Frizzled Related ProteinFrizzled1–10
GFAP	glial fibrillary acidic protein
GSK3	glycogen synthase kinase 3
GLT–1	glutamate transporter 1
Ins (1,4,5)P3	inositol–1,4,5–trisphosphate
JNK	JUN–N–terminal kinase
LRP5/6	low–density lipoprotein receptor–related protein5/6
LRP4	low–density lipoprotein receptor–related protein 4
mEPSCs	miniature excitatory postsynaptic currents
MUSK	muscle skeletal receptor
NMJ	neuromuscular junction
NPCs	neural progenitor cells
NSCs	neural stem cells
PCP	planar cell polarity
PLC	phospholipase C
PTK7	protein Tyr kinase 7
ROR1/2	receptor Tyr kinase–like orphan receptor1/2
RYK	receptor Tyr kinase
SOD1	superoxide dismutase–1
